# Congenital Leptin Deficiency and Leptin Gene Missense Mutation Found in Two Colombian Sisters with Severe Obesity

**DOI:** 10.3390/genes10050342

**Published:** 2019-05-07

**Authors:** Hernan Yupanqui-Lozno, Raul A. Bastarrachea, Maria E. Yupanqui-Velazco, Monica Alvarez-Jaramillo, Esteban Medina-Méndez, Aida P. Giraldo-Peña, Alexandra Arias-Serrano, Carolina Torres-Forero, Angelica M. Garcia-Ordoñez, Claudio A. Mastronardi, Carlos M. Restrepo, Ernesto Rodriguez-Ayala, Edna J. Nava-Gonzalez, Mauricio Arcos-Burgos, Jack W. Kent, Shelley A. Cole, Julio Licinio, Luis G. Celis-Regalado

**Affiliations:** 1 Dexa Diab IPS, Bogotá 110221, Colombia; h.yupanqui1@hotmail.com (H.Y.-L.); maria.yupanquiv@gmail.com (M.E.Y.-V.); 2 Texas Biomedical Research Institute, San Antonio, TX 78227, USA; raul@txbiomed.org (R.A.B.); ernito18@gmail.com (E.R.-A.); edna.navag@uanl.mx (E.J.N.-G.); jkent@txbiomed.org (J.W.K.J.); scole@txbiomed.org (S.A.C.); 3 Facultad de Medicina, Fundación Universitaria Juan N. Corpas, Bogotá 111161, Colombia; monica.alvarez@juanncorpas.edu.co; 4 Genetica Molecular de Colombia, Bogotá 111221, Colombia; oseban87@gmail.com; 5 Facultad de Medicina, Universidad de La Sabana, Chía 53753, Colombia; aidagiraldo92@gmail.com (A.P.G.-P.); alexandraarse09@hotmail.com (A.A.-S.); caritorres147@hotmail.com (C.T.-F.); amgohu@hotmail.com (A.M.G.-O.); 6 NeuRos, Instituto de Medicina Traslacional, Escuela de Medicina y Ciencias de la Salud, Universidad del Rosario, Bogotá 111711, Colombia; claudio.mastronardi@urosario.edu.co; 7 GeniURos, CIGGUR, Instituto de Medicina Traslacional, Escuela de Medicina y Ciencias de la Salud, Universidad del Rosario, Bogotá 111711, Colombia; carlosmrestrepo@gmail.com; 8 Grupo de Investigación en Psiquiatría (GIPSI), Instituto de Investigaciones Médicas. Facultad de Medicina, Universidad de Antioquia, Medellín 050010, Colombia; mauricioarcosburgos@gmail.com; 9 SUNY Upstate Medical University, Syracuse, NY 13210, USA; juliolicinio@gmail.com

**Keywords:** congenital leptin deficiency, novel mutation, extreme obesity, Colombian sisters, *LEP* gene, consanguinity

## Abstract

Background: Congenital leptin deficiency is a recessive genetic disorder associated with severe early-onset obesity. It is caused by mutations in the leptin (*LEP*) gene, which encodes the protein product leptin. These mutations may cause nonsense-mediated mRNA decay, defective secretion or the phenomenon of biologically inactive leptin, but typically lead to an absence of circulating leptin, resulting in a rare type of monogenic extreme obesity with intense hyperphagia, and serious metabolic abnormalities. Methods: We present two severely obese sisters from Colombia, members of the same lineal consanguinity. Their serum leptin was measured by MicroELISA. DNA sequencing was performed on MiSeq equipment (Illumina) of a next-generation sequencing (NGS) panel involving genes related to severe obesity, including *LEP*. Results: Direct sequencing of the coding region of *LEP* gene in the sisters revealed a novel homozygous missense mutation in exon 3 [NM_002303.3], C350G>T [p.C117F]. Detailed information and clinical measurements of these sisters were also collected. Their serum leptin levels were undetectable despite their markedly elevated fat mass. Conclusions: The mutation of *LEP*, absence of detectable leptin, and the severe obesity found in these sisters provide the first evidence of monogenic leptin deficiency reported in the continents of North and South America.

## 1. Introduction

Obesity is a pandemic worldwide and is closely associated with multiple metabolic disturbances including diabetes, hyperlipidemia, nonalcoholic fatty liver disease, hypertension, and cardiovascular diseases, as well as various types of cancer. Undoubtedly, an excess of fat poses both a significant health threat to individuals and a major global public health problem [1]. The increasing prevalence of childhood obesity [2], in particular, signals towards a burden of disease in young and adult individuals with additional healthcare burden in the years to come [3]. Obesity is a heritable disorder [4]. Although most forms of obesity [5] are influenced by both genetic [6] and environmental factors, monogenic obesity is a very rare type of obesity, which is caused by a mutation in a single gene and is usually not significantly affected by the environmental factors, resulting in severe obesity in early childhood [7].

Several genes, such as pro-opiomelanocortin (*POMC*), leptin receptor (*LEPR*), leptin (*LEP*), proconvertase 1 prohormone convertase 1 (*PC1*), and melanocortin 4 receptor (*MC4R*), have been confirmed as harboring mutations that are casual to the onset of monogenic obesity, together accounting for 3–5% of non-syndromic cases [8], although there is evidence that genetic variants in LEP, LEPR, and MC4R explain 30% of severe obesity in children from consanguineous populations [9]. Of note, the most common monogenic form of obesity in humans is due to mutations in *MC4R* [10]. Congenital leptin deficiency (CLD) is a rare human genetic disorder caused by homozygous mutations of the *LEP* gene resulting in severe hyperphagia and early-onset obesity [11]. Most patients described to date have had consanguineous parents [12].

Leptin is secreted by adipose tissue and regulates energy homeostasis, neuroendocrine function, metabolism, immune function and other systems through its effects on the central nervous system and peripheral tissues. Circulating leptin levels are directly in proportion to the amount of body fat, thereby reflecting the status of long-term energy stores. Leptin interacts with a complex neural circuit to control food intake, activating anorexigenic neurons that synthesize pro-opiomelanocortin (POMC) and inhibiting orexigenic neurons that synthesize agouti-related peptide (AgRP) and neuropeptide Y (NPY). In addition to regulating food intake, leptin increases energy expenditure through sympathetic nerve activity [13]. Mutations in the mouse leptin gene lead to severe obesity and, in nearly all cases, very low plasma leptin concentration [14]. However, a homozygous mouse leptin V145E mutant genotype (*LEP^V145EV/145E^*) with high circulating leptin levels has been described [15]. Children with CLD have normal birth weight but rapidly gain weight in the first months of life leading to extreme obesity, impaired satiety and intense hyperphagia. They also develop metabolic and hormonal alterations including hyperinsulinemia, insulin resistance, severe liver steatosis, dyslipidemia, and hypogonadotropic hypogonadism [16].

Leptin, a 167-amino acid protein produced by the *LEP* gene is located on chromosome 7q31.3 [17]. Several mutations of *LEP* associated with CLD have been described in humans to date. These findings have been documented from several countries and specific regions with high rates of consanguinity [12]. Approximately, 80% of patients described in the literature come from Central Pakistan [18]. The first human mutation was reported in two severely obese cousins from a consanguineous UK family of Pakistani origin. This homozygous single base deletion at codon 133 caused a frameshift mutation resulting in a truncated protein and undetectable serum leptin levels [19]. A second mutation was described in three individuals from a consanguineous Turkish pedigree [20]. Mutations have also been found in two individuals from a population inhabiting a small Turkmen mountain village [21], in two children from a consanguineous Egyptian pedigree [22], a child from an Austrian pedigree without known consanguinity [23], in individuals originating from consanguineous Pakistani pedigrees [24], in a child from a consanguineous Indian pedigree [25], and a mutation causal in the heterozygous state, H118L, detected in severely obese Chinese patients [26]. An interesting mutation was found in two siblings, a 9-year-old girl and a 6-year-old boy with severe early-onset obesity and hyperphagia, both homozygous for a c.309C>A substitution in the leptin gene leading to a p.N103K amino acid exchange in the protein and detectable circulating levels of leptin. The p.N103K *LEP* mutation causes obesity due to biological inactivity, but in the presence of high circulating levels of the mutant leptin hormone [27]. Saeed et al. identified in 2015 a novel missense recessive mutation in exon 3, in a 1.6-year-old boy. This mutation is responsible for a substitution of tyrosine in place of cysteine, at residue 117, resulting in impaired protein function (c.350G>A, p.C117Y). The child was severely obese and was clinically leptin deficient. His parents were shown to be heterozygous carriers for this mutation [9]. Recently, a novel, homozygous, missense mutation in exon 3 of the *LEP* gene (C.298G>A) was reported in an infant from northwest India [18]. In this communication, we report a novel homozygous missense mutation [NM_002303.3], c.350G>T [p.C117F] in *LEP* associated with very low serum leptin concentrations, hyperphagia, and early-onset obesity in two severely obese sisters from Colombia born from consanguineous parents.

## 2. Materials and Methods

### 2.1. Ethics Statement, Consent, and Permissions

This study was conducted in accordance with the Declaration of Helsinki and approved by the Institutional Review Board of Sabana University, Chia, Colombia. Written informed consent was obtained from each participant and parents.

### 2.2. Subjects

**Figure 1:**
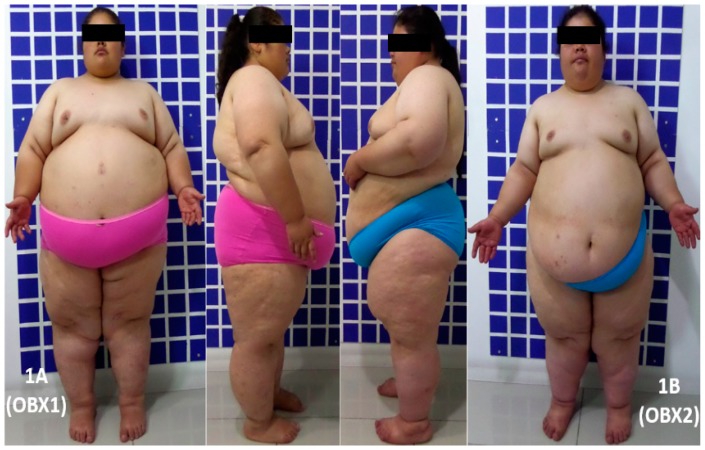
Clinical photographs of two Colombian sisters with congenital leptin deficiency with a newly reported mutation in the leptin gene. OBX1 (**A**) is a 24-year-old female and OBX2 (**B**) is her 21-year-old sister.

**Figure 2:**
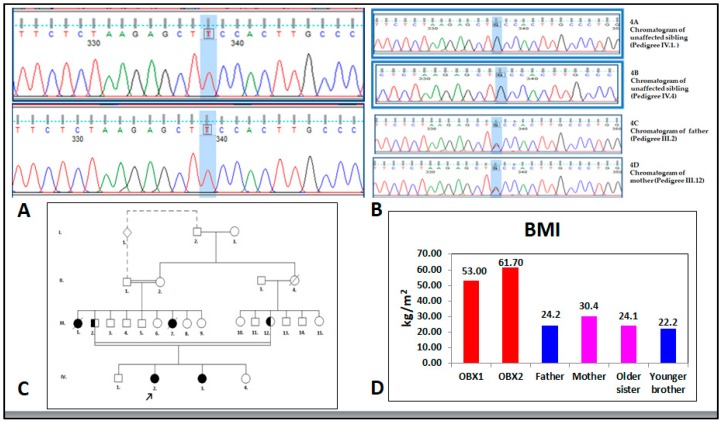
(**A**) DNA sequencing chromatogram indicating leptin (*LEP*) gene homozygous mutation c.350G>T (p.Cys117Phe) in severely obese sisters OBX1 and OBX2. (**B**) DNA sequencing chromatogram indicating *LEP* gene homozygous mutation c.350G>T (p.Cys117Phe) in the father and mother of the severely obese sisters OBX1 and OBX2, but not in their siblings. (**C**) Pedigree of the family. The black arrow indicates sister OBX1 (arrow at IV.2) and sister OBX2 (IV.3). This pedigree includes four generations of a highly inbred family, having at least two identified consanguineous unions and four affected people evaluated by the research group, suggesting an autosomal recessive inheritance pattern. We could assume that one variant is carried by the father and sisters. Unfortunately, we do not have genotyping for any of the grandparents still alive. We could only speculate that either one of the grandparents might carry the contributing variant. (**D**) BMI from the affected sisters, parents, and siblings.

The two extremely obese sisters enrolled (here referred to as OBX1 and OBX2, Figure 1) were identified while attending an endocrinology clinic due to early childhood-onset severe obesity. Clinical histories for each person were compiled, followed by a complete physical examination emphasizing the clinical characteristics observed. A cause of genetic origin was suspected. Pedigree information was compiled and blood samples were taken for DNA sequencing chromatograms (Figure 2A), laboratory tests, including genetic and hormone analysis, and diagnostic images.

### 2.3. Sequencing of Leptin Genomic DNA

A white blood cell-rich concentrate, the buffy coat, was obtained by centrifuging the unclotted blood samples using a standard blood bank centrifuge. Genomic DNA was extracted from these blood samples (peripheral blood mononuclear cells) taken from both sisters, their parents, and two brothers, using Agencourt Genfind v2 (Beckman Coulter, Life Sciences, Indianapolis, USA) on a Biomek FXP (Beckman Coulter, Life Sciences) liquid handling robot. DNA was quantified using QuBit dsDNA HS Assay and Qubit Fluorometer (Life Technologies, Thermo Fisher Scientific, Waltham, MA, USA). NGS sample preparation and enrichment was performed using Illumina’s Nextera XT kit according to manufacturer’s recommendation. Paired-end Illumina MiSeq sequencing was performed on an Illumina MiSeq device (Illumina Inc., San Diego, CA, USA) for a total of 600 cycles (300 cycles for each read. A dual index barcode sequence read of 6 bases per index were added to each sample as a unique identifier for a total of 12 cycles) according to the manufacturer’s instructions, for a next-generation sequencing (NGS) panel involving genes related to severe obesity: *LEP* (NM_000230.3), *LEPR* (NM_002303.5), *PPARG* (NM_015869.4), *MC4R* (NM_005912.2), *PCSK1* (NM_00439.4), and *POMC* genes (NM_001035256.1). All sequencing work was performed in Bogota, Colombia.

### 2.4. Bioinformatics Analysis to Identify Point Mutations, Small Deletions, Insertions and Alterations

RTA (Real Time Analyses) software version 1.13.48 took data from the sequence raw images to Illumina’s BCL format binary files for base calls and quality scores. BCL files were converted to FASTQ files dimultiplexed by Illumina’s CASAVA pipeline version 1.8.2. We used the GATK (Genome Analysis Toolkit, Broad Institute, Cambridge, MA, USA) structured programming framework to obtain rich sets of data access patterns, base quality score recalibration, indel realignment, duplicate removal, and SNP and INDEL discovery [28]. To validate detected mutations, the *LEP* gene exon 3 encoding region (ENST00000308868.4) was amplified by PCR. The amplicon was purified by alkaline phosphatase and exonuclease I, and directly sequenced all along its length by the Sanger method [29]. Each person’s sequence was compared to the reference wild type sequence. Nucleotide numbering was begun at the first adenine (A) of the ATG start codon in position 1. In summary, the targeted amplicons of each sample were sequenced on MiSeq in 300 bp paired-end reads. The potential causative variants identified by targeted sequencing were confirmed by Sanger sequencing via the 3730 × l DNA Analyzer (Life Technologies, Carlsbad, CA, USA).

## 3. Results

Case History 1. Our first report is a 24-year-old female, referred to as OBX1 (Figure 1A). She was first seen in the practice setting at age 9 for early childhood-onset morbid obesity, primary amenorrhea, and acne on the chest. She was a second child, born after a 38-week uneventful pregnancy and delivery; normal weight and height at birth. There was no presence of failure to thrive during the first two years. Progressive weight gain was observed reaching a BMI of 40 kg/m^2^ at 14 years-old. Bariatric surgery (sleeve gastrectomy) was performed when she was 16 years-old, after she reached 120 kg weight and a BMI of 53 kg/m^2^. Her weight decreased to 100 kg following surgery, but it progressively increased to 141 kg over the last 5 years in spite of a dietary approach including a low fat and low sugar diet, along with a 60-min walk daily. Her parents are first cousins and her maternal and paternal grandmothers are sisters (Figure 2B,C). Her father weighs 70 kg, his height is 170 cm (BMI = 24.2 kg/m^2^), and he is 52 years old. Her mother weighs 72 kg, her height is 154 cm (BMI = 30.4 kg/m^2^), and she is 48 years old. Her older sister weighs 68 kg, her height is 168 cm (BMI = 24.1 kg/m^2^), and she is 27 years old. Her younger brother weighs 50 kg, his height is 150 cm (BMI = 22.2 kg/m^2^), and he is 17 years old (Figure 2D). A paternal aunt who died aged 29 years old suffered from extreme obesity, primary amenorrhea, diabetes mellitus, and leukemia; another paternal aunt developed rapid-onset obesity and died at one year of age, having been born with a healthy appearance. Physical examination revealed her blood pressure was 109/72 mm Hg, her heart rate was 69 bpm, her respiratory rate was 18 rpm, her body temperature was 36.1 °C, her weight was 143.9 kg, her height was 149.7 cm, her waist circumference was 152 cm, and her BMI was 64.2 kg/m^2^. She had severe obesity, normocephaly (without cognitive or motor deficit), average intelligence, left eye moderate strabismus, mild hirsutism (Ferriman and Gallwey score 11) [30], hair in the maxillary region, mild acantosis nigricans, symmetrical thorax covered with abundant acne, normal heart rhythm (no murmurs), abundant panniculus adiposus, Tanner stage breast development V, female genitals with Tanner stage V pubic hair [31], abundant lower limb telangiectasia, pain on mobilizing the left knee, small hands and feet, clinodactyly, and nail hypoplasia (Figure 1A). Her clinical biochemistry panel was 19.9 pg/mL for estradiol (12.5–166 pg/mL reference value (RV), 6.58 mUI/mL for FSH (5.8–21 mUI/mL RV), 2.71 mUI/mL for LH (1.1–11.6 mUI/mL RV), 4.89 mUI/mL for prolactin (1.39–24.2 mUI/mL RV), 0.84 mUI/mL for TSH (0.4–4 mUI/mL RV), and a nuclear magnetic resonance of the hypophysis was reported normal. Her last report in fasting cardiovascular risk and metabolic clinical chemistry was glucose 77 mg/dL, insulin 19.9 µIU/mL, HDL cholesterol 43 mg/dL, triglycerides 327 mg/dL, and HbA1c 6.6%.

Case History 2. Our second report is a 21-year-old female OBX2 (Figure 1B) (IV.3 in the pedigree chart, Figure 2C). She was first seen in the practice setting at age 6. She is the third child of the nuclear family and younger sister of OBX1. She has consulted for early childhood-onset morbid obesity and primary amenorrhea; she had hypertriglyceridemia, insulin resistance, and *acantosis nigricans*. She was receiving 600 mg of gemfibrozil and 850 mg of metformin as daily treatment. She was born after a 38-week pregnancy involving non-hospital vaginal delivery; somatometry at birth was not obtained, although the mother stated that her weight and height at birth were normal and similar to that of her sister. Development and thriving was similar to her sister. Physical examination revealed her blood pressure was 106/70 mm Hg, her heart rate was 93 bpm, her respiratory rate was 21 rpm, her body temperature was 37.5 °C, her weight was 133.45 kg, her height was 147.1 cm, her waist circumference was 144 cm, and her BMI was 61.7 kg/m^2^. She had severe obesity, a normal neurological exam, average intelligence, a severe left eye strabismus, mild hirsutism (Ferriman and Gallwey score 11), acantosis nigricans on the neck, heart exam revealed no alterations, mild acne on the chest, abundant abdominal and body panniculus adiposus, Tanner breast development stage V, normal external female genitals, Tanner stage V [31], and telangiectasias in both lower limbs, clinodactily, nail hypoplasia, and bilateral fifth toe hypoplasia. Her clinical biochemistry panel was 19.2 pg/mL for estradiol (12.5–166 pg/mL RV), 4.17 mUI/mL for FSH (5.8–21 mUI/mL RV), 2.93 mUI/mL for LH (1.1–11.6 mUI/mL RV), 5.37 mUI/mL for prolactin (1.39–24.2 mUI/mL RV), 2.26 mUI/mL for TSH (0.4–4 mUI/mL RV) and a nuclear magnetic resonance of the sella turcica was reported normal. Her last report in fasting cardiovascular risk and metabolic clinical chemistry was glucose 89 mg/dL, insulin 6.0 µIU/mL, HDL Cholesterol 51 mg/dL, triglycerides 203 mg/dL, and HbA1c 5.4%.

**Figure 3:**
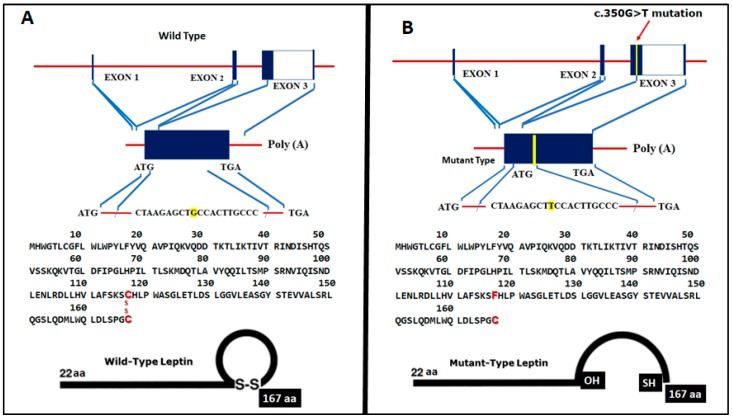
(**A**) Genomic structure of the *LEP* gene (wild type), showing all exons, oligonucleotides, and amino acid sequence. (**B**) Novel mutation in *LEP* gene–Leptin mRNA (NM_000230.2): c.350G>T (p.C117F) in the severe obese sisters (in-frame transversion of G to T) mutation in nucleotide 350 in the encoding sequence, guanine (G) replaced by thymine (T), causing Cys replacement by Phe in amino acid 117, with lack of intrachain disulfide bond.

Genetics. Sequencing of the genes *LEP*, *LEPR*, *PPARG*, *MC4R*, *PCSK1*, and *POMC* revealed the presence of three polymorphisms in *LEP* not associated with the patients’ clinical picture: c.198G>C (14% allele frequency), c.668A>G (41%), and c.3057G>A (46%), (all missense); such variants were considered normal polymorphisms due to their high frequency in the population [32]. Furthermore, two missense variations were considered neutral for the *PCSK1* gene (NM_00439.4) located in c.2069G>C (25% allele frequency) and c.661A>G (1.7%) (data not shown). A relevant finding was the identification of a novel *LEP* gene variant (NM_002303.3), C.350G>T (p.C117F) that was present in the homozygous state in both sisters (Figure 3B). This variant occurred within exon 3 in a functional part of the leptin protein, which has been highly conserved during evolution [33].

Leptin profile of the sisters. The range reference value (RV) of leptin circulating levels for females (with normal BMI) is 3.7–11.1 ng/mL, as provided by the assay manufacturer. This range for typical obese individuals will directly increase according to the mass accumulation of their adipose tissue. The sister’s leptin levels were below the detection limit of the kit.

## 4. Discussion

There is ample evidence [34] documenting a significant genetic contribution to the regulation of body weight [35] and childhood obesity [36]. Moreover, about 5% of all children with severe obesity may have monogenic obesity caused by mutations in one of the several genes involved in the regulation of appetite and body weight, including the rarer syndromic forms of early-onset obesity [37] such as Bardet–Biedl syndrome (BBS) [38], Prader–Willi syndrome [39] and Beckwith–Wiedemann syndrome [40]. A treatable form of monogenic obesity is due to homozygous mutations in the *LEP* gene leading to recessively inherited congenital leptin deficiency [19].

In both mice [41] and humans, congenital leptin deficiency is associated with a normal birth weight followed by the rapid development of severe obesity associated with hyperphagia and impaired satiety [42]. It is currently recommended that children with normal weight at birth experiencing accelerated weight gain in the first months of life leading to extreme obesity and symptoms such as impaired satiety, intense hyperphagia, and food-seeking behavior should be tested for CLD [43]. CLD is a treatable monogenic form of obesity [44]. Leptin replacement may also be a viable treatment for congenital generalized or acquired generalized lipodystrophy [45]. Recombinant human leptin (RHL) has been used as substitution therapy for leptin deficiency, via a once daily subcutaneous injection [46]. The effects have been studied extensively in humans suffering such deficit [44]. The form of leptin currently available to humans is recombinant methionyl human leptin, or metreleptin, which has been recently approved by the US Food and Drugs Administration (FDA) for treating generalized congenital or acquired lipodystrophy [47].

We found two related females with extreme obesity. The two subjects, here referred to as OBX1 and OBX2 (Figure 1), are sisters within a highly consanguineous family of Colombian origin (Figure 2C). Their ethnical origin is from the Muisca [48] indigenous group of the Altiplano Cundiboyacense, Colombia [49]. The majority of mutations of *LEP* have been associated with high rates of consanguinity [12], as in the present case. The prevalence of consanguinity in Colombia is the third highest in South America after Brazil and Venezuela at 1.30% [50]. Three regions in Colombia (Santander, Boyacá, and Antioquia) have high levels of inbreeding. In particular, the region of Boyacá, from which these patients were recruited, is the location where inbreeding occurs especially frequently due to geographical and economic isolation [51]. Saeed et al. [12] have concluded that obesity is the most heritable disorder. However, only less than 5% of patients with extreme obesity are caused by single gene mutations. In contrast, in highly consanguineous families such as the one of the two Colombian sisters, a striking amount of cases with extreme obesity due to monogenic mutations (up to 30%) can be identified.

Although of normal weight at birth, both sisters suffered from severe, intractable obesity from an early age. The sisters had no additional clinical features to suggest that they might have a pleiotropic genetic syndrome associated with obesity, such as Prader–Willi syndrome. We detected a novel mutation Leptin mRNA (NM_000230.2): c.350G>T (p.C117F) in the severe obese sisters, speculating that this homozygous mutation in their *LEP* gene to be causal to their monogenic obesity. This missense mutation occurred in nucleotide 350 in the gene’s encoding sequence, with guanine (G) being replaced by thymine (T), in turn causing a cysteine (Cys) replacement by phenylalanine (Phe) in amino acid 117, thereby affecting an amino acid residue that has been conferring stability to the tertiary structure of the protein (Figure 3A,B). The Cys in leptin at position 117 is responsible for the intramolecular disulfide bond. The formation of an intramolecular disulfide bridge is necessary for normal processing and secretion of leptin [52]. To our knowledge, this is the first time that a putatively causal *LEP* mutation has been reported in the continents of North and South America.

It appears that the lack of intrachain disulfide bond impairs leptin secretion. Crystallization of leptin has shown that this protein contains an intrachain disulfide bond [53]. Disulfide bonds play an important role in the folding, stability, and efficiency of secretion of proteins and regulate their retention time in the endoplasmic reticulum [54]. Disulfides inhibit protein aggregation by producing more compact folding intermediates [55]. It has been demonstrated that the lack of disulfide bond is itself sufficient to impair leptin secretion resulting in the accumulation of macromolecular aggregates in the cell. Intramolecular disulfide bonds interacting with mutagenesis of cysteines lead to protein misfolding, aggregation, and impaired secretion (Figure 3A,B) [52]. Although we have no direct evidence that disulfide bond formation is impaired in the *LEP* C350G>T [p.C117F] mutant found in the sisters, the similarity of the behavior of cysteine mutants suggests that impaired formation of disulfide bond could be a potential mechanism for the lack of secretion of the occurring *LEP* mutant.

The clinical picture was quite similar in both sisters: severe obesity, primary amenorrhea, insulin resistance, hypertriglyceridemia, acantosis nigricans, and hyperphagia. Although a formal assessment of appetite was not conducted, we were able to document a history of marked hyperphagia, with both sisters noted from early infancy to be constantly hungry, demanding food continuously and eating considerably more than their other siblings. Detailed assessment of resting metabolic rate and total daily energy expenditure was not performed in these sisters, although their mean body temperatures were within the normal range. Farooqi et al. have reported high rates of childhood infection and atopic disease due to abnormalities of T-cell number and function in children with CLD [56]. The clinical history of these two Colombian sisters did not report frequent infections. Early reports of leptin deficiency caused by a missense mutation in human subjects have shown at physical examination scarce hair in the pubic and axillary area [57]. Here, both sisters presented mild hirsutism (Ferriman and Gallwey score 11) and female genitals with Tanner stage V pubic hair. Hirsutism represents a primary clinical indicator of androgen excess. The most common endocrine condition causing hirsutism is polycystic ovary syndrome (PCOS) [58]. Idiopatic hirsutism (IH) [59], the second most common cause of hirsutism after PCOS, is considered when hirsutism is associated with normal ovulatory function and normal circulating serum androgen concentrations. The pathogenesis of IH is still unclear, although increased activity of peripheral 5-α reductase enzyme [60], androgen receptor gene polymorphism [61], and increased sensitivity of hair follicles to androgens have been proposed [62].

To examine whether homozygosity for this in-frame transversion of G to T mutation was associated with abnormalities in circulating leptin, serum leptin levels were measured in both sisters and found to be below the limits of detection. Because in most obese subjects, serum leptin levels were high and correlated with the body mass index (BMI) and the percentage of body fat, the finding of undetectable levels of serum leptin in these two extremely obese children was significant. Our observations indicate that their severe obesity is due to a congenital deficiency in the production of leptin. Fifty seven homozygous and heterozygous mutations in the *LEP* gene have been described in humans to date: 1.5% likely benign, 18% benign, 1.5% likely pathogenic, 21% pathogenic, and 58% unknown [63]. Two of the mutations have been described as being located proximal to the novel mutation reported here, one on nucleotide 360 Leptin mRNA (NM_000230.2:c.*360G>A) [37], and another one on nucleotide 350 (NM_000230.2:c. 350A>T) [26]. One limitation of our study was that a functional study of this novel mutation Leptin mRNA (NM_000230.2): c.350G>T (p.C117F) was not carried out to elucidate the mechanism of the disease.

## 5. Conclusions

We report here findings from two Colombian sisters with congenital leptin deficiency and early-onset severe obesity born from parents with known consanguinity. The molecular diagnosis of this rare genetic disorder is important due to the possibility of treatment with recombinant leptin.
